# Silencing the FABP3 gene in insulin-secreting cells reduces fatty acid uptake and protects against lipotoxicity

**DOI:** 10.1007/s00592-024-02325-x

**Published:** 2024-07-04

**Authors:** Ayman Hyder, Basma Sheta, Manar Eissa, Jürgen Schrezenmeir

**Affiliations:** 1 Faculty of Science, Damietta University, New Damietta, 34517 Egypt; 2 Faculty of Medicine, Johannes Gutenberg University, Mainz, Germany

**Keywords:** Insulin secretion, Beta cell, Fatty acid, Binding protein, Inflammation, Apoptosis

## Abstract

**Background:**

Long-term exposure of pancreatic islets to fatty acids (FAs), common in obesity, metabolic syndrome, and type 2 diabetes, leads to a compensatory hyperactivity followed by inflammation, apoptosis, dysfunctional beta cells, and results in insulin dependence of the patient. Restriction of fatty uptake by islet beta cells may protect them from lipotoxicity.

**Purpose:**

Pancreatic islet beta cells express the fatty acid binding protein 3 (FABP3) to bind FAs and to orchestrate lipid signals. Based on this, we investigated whether downregulation of FABP3, by *Fabp3* silencing, might slow lipid metabolism and protect against lipotoxicity in insulin-secreting cells.

**Results:**

Neither *Fabp3* silencing, nor overexpression affected the glucose-stimulated insulin secretion in absence of FAs. *Fabp3* silencing decreased FA-uptake, lipid droplets formation, and the expression of the lipid accumulation-regulating gene *Dgat1* in Ins1E cells. It reduced FA-induced inflammation by deactivation of NF-κB, which was associated with upregulation of IκBα and deactivation of the NF-κB p65 nuclear translocation, and the downregulation of the cytokines ILl-6, IL-1β, and TNFα. Ins1E cells were protected from the FA-induced apoptosis as assessed by different parameters including DNA degradation and cleaved caspase-3 immunoblotting. Furthermore, FABP3 silencing improved the viability, *Pdx1* gene expression, and the insulin-secreting function in cells long-term cultured with palmitic acid. All results were confirmed by the opposite action rendered by FABP3 overexpression.

**Conclusion:**

The present data reveals that pancreatic beta cells can be protected from lipotoxicity by inhibition of FA-uptake, intracellular utilization and accumulation. FABP3 inhibition, hence, may be a useful pharmaceutical approach in obesity, metabolic syndrome, and type 2 diabetes.

**Supplementary Information:**

The online version contains supplementary material available at 10.1007/s00592-024-02325-x.

## Introduction

Pancreatic islet beta cell lipotoxicity refers to the excessive accumulation of FAs within cells leading to their dysfunction, death by apoptosis [1]. There is a strong correlation between lipotoxicity-induced beta cell dysfunction and death and the development of obesity, metabolic syndrome, and progression of type 2 diabetes [2]. These disorders are linked with increased oxidative stress, ceramide accumulation, and activation of stress responses [3]. In pancreatic islets of Langerhans, FAs have a dual effect [4], since the short-term exposure stimulates insulin secretion, while the long-term exposure to FAs increases basal insulin release and reduces glucose-stimulated insulin secretion (GSIS). It also causes islet beta cell failure and apoptosis, especially when glucose levels are elevated. The contribution of lipotoxicity to deterioration of insulin secretion and peripheral insulin resistance, and its association with the increased obesity prevalence, metabolic syndrome, and type 2 diabetes were early reported [5]. There is evidence that the 5- to 10-fold increment in islet lipid content that occurs in the prediabetic phase triggers the compensatory beta cell hyperplasia and hyperinsulinemia. Further elevation in islets’ fats reverses the preceding compensatory changes and initiates beta cell dysfunction and diabetes [6].

The transport and metabolism of the insoluble FAs are regulated by membrane-associated and cytosolic proteins that bind and transport FAs. There are ten distinct fatty acid binding proteins (FABPs), with tissue-specific expression patterns [7]. These intracellular FA transport proteins participate in lipid metabolism by binding FAs, regulating gene expression, and orchestrating lipid signals. Pancreatic islets mostly express the heart/muscle type (FABP3) to bind, traffic and metabolize FAs [8]. FABP3 is a 14–15 kDa protein abundantly expressed in the muscle, heart, and brain [9]. It binds to long-chain FAs and transports them to different subcellular compartments for lipid storage and metabolism [10]. In addition to its lipid metabolic role, FABP3 has other important functions on signaling transduction and transcriptional regulation [11, 12]. For example, overexpression of FABP3 upregulated the phosphorylation of MAPK signaling pathway and decreased phosphorylated Akt levels, which may account for the augmentation of apoptosis after myocardial infarction [13]. Also, FABP3 is involved in the control of DNA methylation of the Gad67 promoter and activation of GABAergic neurons in the anterior cingulate cortex [14].

In different cell systems, variable FABPs were reported to boost FA uptake and triglycerides (TG) accumulation. Manipulation of different FABP family members has been reported to control FA uptake and intracellular lipid accumulation. In mouse liver, knocking down the *fabp1* gene reduced hepatic TG accumulation and FA uptake [15]. In mouse brain, *Fabp3* gene knockout has been reported to decrease arachidonic PUFA uptake and alter the phospholipid composition [16]. In L cells (a fibroblast cell line), overexpression of FABP1 and 2 increased phospholipid content and altered the fatty acid composition of phospholipids [17]. Similarly, knocking down *fabp4* in mouse prevented the progression of insulin resistance and atherosclerosis [18], whereas its knocking out improved osteoarthritis induced by HFD in mice [19].

Therefore, inhibition of FABP3 in the pancreatic islets may protect against TG accumulation and the consequent impaired insulin secretory function and beta cell death. The main hypothesis in this work is that the knocking down *Fabp3* in insulin-secreting cells will reduce FA uptake and the resultant lipotoxicity and prevent apoptosis of insulin-producing cells. The limited expression pattern of FABPs [20] suggests a quite specific action on islet beta cells. Consequently, the aim of the present work was to investigate the effect of FABP3 manipulation by silencing or overexpression on fatty acid uptake into beta cells and their inflammatory response upon FA exposure.

## Materials and methods

### Tissue culture

The rat insulin-secreting beta cell line Ins1E cells [21] were cultured as published before [8]. Pancreatic islets were isolated as described before [22–24] from control or 16 w-high-fat fed male rats. The fat content in the control diet was 4.5%, while that in the high-fat diet was 40% [25]. Ins1E cells (passages 30–50) and islets were cultured at 37 °C with 5% CO_2_ in RPMI-1640 Glutamax medium supplemented with 10% fetal calf serum, 10 mmol/L Hepes, 50 μmol/L 2-mercaptoethanol, 1 mmol/L sodium pyruvate, 100 IU penicillin/ml and 100 μg streptomycin/ml. For the different experiments, cells were cultured in 6-well plates until reaching 80–90% confluence.

For the preparation of fatty acids [26], a stock solution was prepared by dissolving FAs in ethanol to a final concentration of 1 mol/L. Aliquots of this stock solution were dissolved in 10% fatty acid-free bovine serum albumin (Merck) solution in additive-free RPMI medium) to a concentration of 10 mmol/L by incubation in ultrasonic bath. The BSA-bound solution was then diluted in culture media. Cells were fasted for 2 h in supplementation-free RPMI1640 medium before different incubations.

### Cloning of FABP3

The method cloning using the plasmid pCAGGS was reported before [27, 28]. To extract and clone the full-length coding sequence of FABP3, RNA was isolated from rat muscle. The following oligonucleotide primers were designed after examination the sequences for the restriction enzymes (Xho1 and EcoR1) that neither cut within the FABP3 nor the pCAGGS vector sequences. The forward primer sequence was 5′-CTG***GAATTC***ATGGCGGACGCCTTTGTC-3′ which has a restriction site for ***EcoR1***, and the reverse primer was 5′-CAG***CTCGAG***TCACGCCTCCTTCGTAAG-3′ with a restriction site for ***Xho1***. The PCR products were ligated into the vector (T4-Ligase Rapid-Kit (Fermentas, Thermo Fisher Scientific), and clones with the proper full-length FABP3 inserts were selected for sequencing. The cloned FABP3 was identically aligned to the genomic sequences.

### Transfection for FABP3 silencing/overexpression

Ins1E cells were seeded in 24-well plates at a density of 4 × 10^4^ cells per well in 1 ml of culture medium and transfections performed at 85% cell confluence. Prior to transfection, TurboFect (Fermentas, Thermo Fisher Scientific) and MEM (Invitrogen) were mixed and incubated at room temperature for 20 min. 100 ng DNA of the FABP3 construct was added to the prepared transfection medium in a ratio of 3 µl transfection medium to 1 ng DNA as recommended by the manufacturer. Control samples were treated similarly and transfected with the empty vector (pCAGGS). After 48 and 72 h post transfection, RNA was isolated, and reverse transcribed to cDNA. FABP3 gene overexpression was confirmed using quantitative RT-PCR and protein Western blot.

For the small interfering (siRNA) experiments, the On-Target plus smart siRNA pool (Dharmacon L-100472-01-0010, ThermoFisher Scientific) that targets 4 different sequences was applied to silence FABP3. A non-targeting scrambled sequence (siScr) was applied as a negative control. Ins1E cells were reverse transfected with the siRNA pool using siPORT NeoFX Transfection Reagent (Invitrogen). siRNA was prepared in Opti-MEM serum-free medium (Gibco) by mixing 2 ul of the transfection reagent with 5 nM siRNA at RT for 10 min. Ins1E cells (4 × 10^4^ cells per well) were cultured in 24-well plates containing siRNA transfection reagent complexes, to allow for transfection to occur during initial cell adherence. The medium was changed after 24 h by the ordinary culture medium and incubated for additional 24 and 48 h to allow cell recovery. RNA was extracted and reverse transcribed to cDNA. FABP3 gene knockdown was confirmed by quantitative RT-PCR and protein Western blot. Confirmation and efficiency of transfections are shown in the supplementary figure S1.

### Quantitative real-time PCR

Total RNA was extracted and 2 μg RNA was reverse transcribed to first strand complementary DNA as reported before [29]. Quantitative RT-PCR analysis was performed on a quantity of cDNA that corresponded to 20 ng RNA. Quantitative PCR analysis was performed using SYBR Green. The thermal cycling program was 10 min at 95 °C for enzyme activation, denaturation for 15 s at 95 °C, 60 s annealing at 60 °C. A dissociation curve was run for each product to verify the absence of primer dimers or nonspecific products. Different primer pairs used in the present study for studied genes are listed in the supplementary Table S1. To normalize expression data, β-actin was used as a housekeeping and internal control gene. Relative quantification was performed by ΔΔCt method. Data were presented as fold change from the control, whose values were considered as 1.

### Western blotting

Protein was extracted from cells with the aid of tissue lysis buffer (50 mmol/L Hepes, pH 7.5, 150 mmol/L NaCl, 10% glycerol, 1% Triton X-100, 1.5 mmol/L MgCl_2_, 1 mmol/L EGTA, protease cocktail (Roche, one tablet/10 ml final buffer volume). Protein SDS-PAGE electrophoresis was performed using Pierce protein gels (Thermo Scientific) and blotted onto a PVDF membrane (Santa Cruz Biotechnology). The membrane was probed with the following antibodies: beta actin (Santa Cruz), cleaved caspase 3 (Cell Signaling), FABP3 (Hycult), histone H3 (Abcam), NF-κB p65 (Dianova). Detection of proteins was carried out by incubation with appropriate HRP-conjugated secondary antibodies and detected by ECL detection reagents (Thermo-Fisher Scientific).

### Fatty acid uptake and lipid droplet assay

The QBT Fatty Acid Uptake and lipid droplet fluorescence Assay Kit (Molecular Devices, product # R8132), which employs a BODIPY-dodecanoic (lauric) acid fluorescent fatty acid analog. For kinetic FA uptake reading, Ins1E cells of different treatments were seeded in 96-well plates with 10^4^ cells per well in 100 µl of complete RPMI medium and incubated at 37 °C for 24 h. After washing, cells were incubated in serum-free medium for 1 h., which was replaced at the end of the incubation time with 100 µl of the kit fatty acid loading buffer. Real-time uptake kinetic readings in live cells were started immediately with a fluorescence plate bottom reader. For the end point reading mode, cells were incubated for 1h before reading. For lipid droplet imaging, plates were incubated overnight (37 °C, 5% CO_2_) before examination with an inverted fluorescence microscope.

### MTT assay

Ins1E cells were seeded in 96-well plates with 5 × 10^4^ cells per well. Cells of different treatments were incubated with 0.1–0.5 mmol/L palmitic acid for 24–72 h. Cell viability was determined by the 3-[4,5-dimethylthiazol-2-yl]-2,5-diphenyltetrazolium bromide (MTT, Sigma) according to the manufacturer instructions. Cell culture supernatants were aspirated from plates and MTT solution (1 mg/ml) was added. Plates were incubated with MTT for 30 min at 37 °C. Dimethyl sulfoxide (DMSO) was added to dissolve the formazan crystals, and the absorbance was measured at 570 nm, with a correction at 690 nm.

### DNA fragmentation assay

Cytoplasmic histone-associated DNA fragments were measured by the Cell Death Detection ELISA kit (Roche, Merck) following the manufacturer instructions. INS-1E cells were transfected for FABP3 overexpression or silencing and treated on the second day with 0.5 mmol/L of palmitic acid in the presence of 5.6 mmol/L glucose for 24 h. Cells were lysed as described by the kit manufacturer. After centrifugation, the supernatant was added onto the anti-histone-coated microplate, which was incubated for 90 min at RT then washed. Conjugation solution was added, incubated for 90 min, and washed. The color was developed by addition of ABTS (2,2′-azino-di-[3-ethylbenzthiazoline sulfonate]) substrate solution (1 mg/ml). After shaking incubation for 20 min the absorbance was measured at 405 nm.

### Insulin secretion

At the end of the different treatments, cells were washed and incubated for 2 h with KRB solution containing 2.8 mmol/L glucose (basal) followed by incubation with medium containing 16.7 mmol/L glucose (stimulatory). All incubation media were kept in − 20 °C until insulin secretion was assessed. Insulin was quantified using the rat insulin ELISA kit (DRG diagnostics, Germany, cat. no. EIA-2048).

### Statistical analysis

Data were presented as the statistical mean ± SEM (standard error of mean) of N = 4 independent experiments. In measurements of some parameters, where N = 3 was mentioned, samples from only 3 experiments were processed. For experiments including Ins1E cells, used cells were from different passages ranging from 30 to 50. Statistical analyses between different groups were examined by one-way analysis of variance (ANOVA). Student’s t test was used to compare the difference between 2 groups and as a post hoc test whenever ANOVA was significant. A *p* value of < 0.05 was considered statistically significant in all analyses.

## Results

### FABP3 is involved in FA-stimulated functions of insulin-producing tissue

**Fig. 1:**
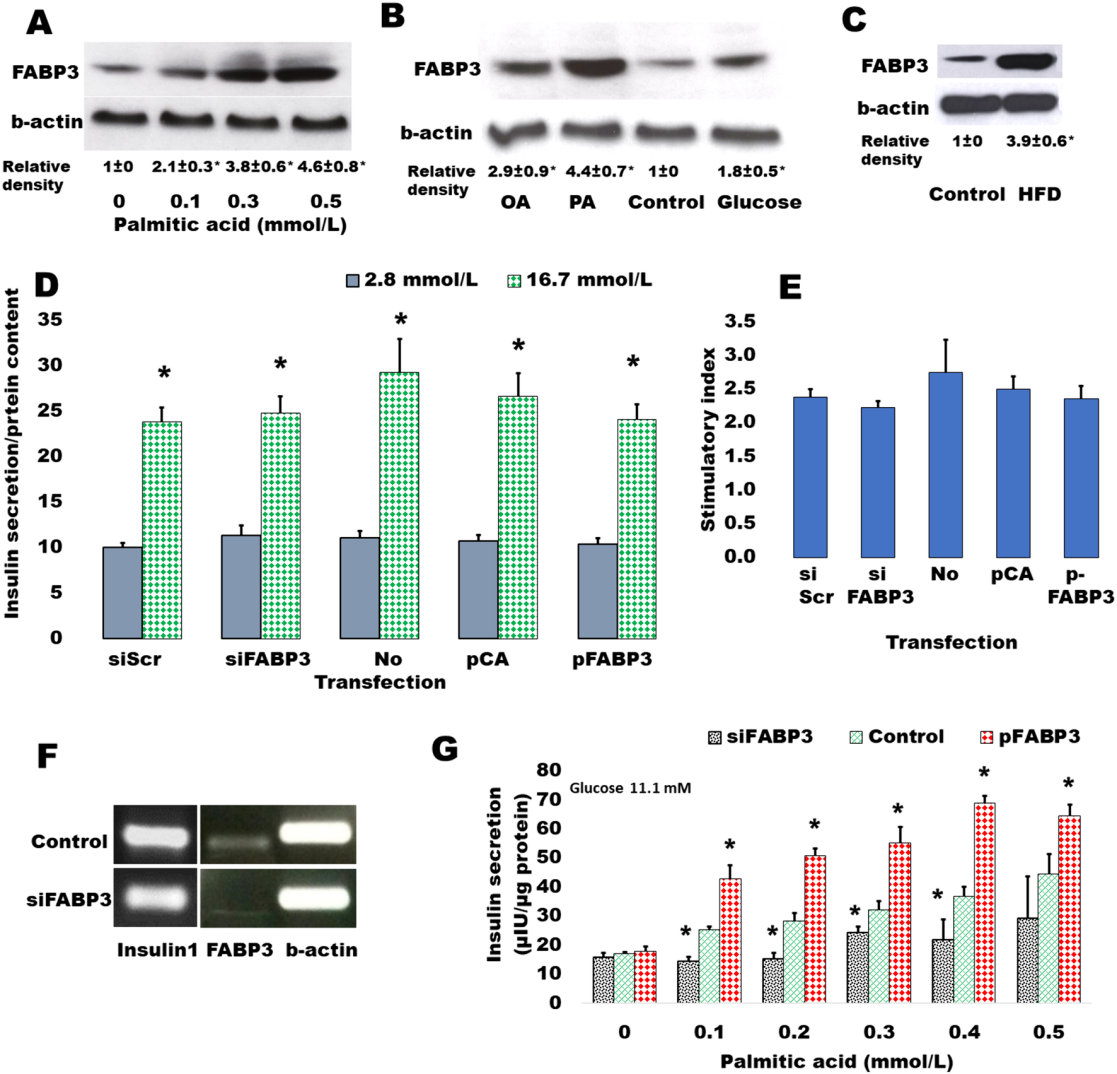
Contribution of FABP3 in insulin-secreting cell function. **A** Palmitic acid induced the expression of the FABP3 protein in a concentration-dependent manner. Rat islets were isolated and cultured for 16 h with different concentrations of palmitic acid. Islet lysates were processed for western blot analysis of FABP3 content. **B** Different fatty acids and glucose variably induced the production of FABP3. Rat pancreatic islets were incubated for 16 h with 0.5 mmol/L of either palmitic (PA), oleic acid (OA) or 16.7 mmol/L glucose. **C** High fat diet increases the islet content of FABP3. Pancreatic islets were isolated from rats that were fed a high-fat diet (HFD) for 16 weeks. Immunoblotting was performed using FABP3 antibody and compared with the blotting of beta-actin as a housekeeping control protein. Quantitative data of western blot images (**A**–**C**) (N = 3) are presented as means ± SEM of relative densities. The “*” denotes a significantly different value from the control value (post hoc t-test after ANOVA *p* < 0.05). **D** Glucose-stimulated insulin secretion (GSIS) is neither affected by FABP3 overexpression nor silencing. Ins1E cells were transfected by either FABP3 siRNA targeting 4 different sequences for silencing FABP3 (siFABP3), non-targeting scrambled sequence (siScr) as a negative control, empty pCAGGS plasmid (pCA), or FABP3-loaded pCAGGS plasmid (pFABP3). Transfected cells were exposed for 2 h to either basal (2.8 mmol/L) or stimulatory glucose concentrations (16.7 mmol/L). The difference between basal and stimulated insulin secretions was significant for all cultures (t-test). No statistical differences were observed among basal groups or stimulated groups (ANOVA). The sub-figure is a summary of 4 separate experiments using cells from different passages. **E** FABP3 silencing or overexpression did not affect the glucose stimulatory index. This index is presented as means ± SEM of the ratios of stimulatory/basal secretions of the previous experiments. No statistical differences were observed among different groups (ANOVA). **F** Knocking down FABP3 gene did not affect the expression of insulin gene in Ins1E cells as observed by standard endpoint PCR. **G** FABP3 gene silencing attenuated, and overexpression provoked palmitate-induced insulin secretion in Ins1E cells. Cells of different transfection groups were incubated for 2 h with 11.1 mmol/L glucose and different concentrations of palmitate. Data are presented as means ± SEM of N = 4 experiments. Statistical analysis: ANOVA *p* < 0.01; the “*” denotes a significantly different value from the control value

The dependence of FABP3 expression in insulin-secreting cells from FA supply was first investigated (Fig. 1). Rat islets were incubated overnight with ascending concentrations of palmitic acid ranging from 0 (control) to 0.5 mmol/L (Fig. 1A). It was found that FABP3 production significantly increased with palmitic acid concentration. In confirmation with the previous study [8], the production level of FABP3 protein in response to different stimulators was variable (Fig. 1B). Palmitic acid increased the cellular level of FABP3 more than oleic acid. Glucose also stimulated the production of FABP3 in islet cells. The results were confirmed in vivo*:* a significantly higher level of FABP3 was found by western blotting in protein lysates of pancreatic islets isolated from rats that were fed on a high-fat diet for 16 weeks (Fig. 1C).

To investigate the role of FABP3 in the glucose-stimulated insulin secretion (GSIS) process, we exposed Ins1E cells, in which FABP3 gene was down-knocked or overexpressed, to low (2.8 mmol/L) and high (16.7 mmol/L) glucose. The results revealed that glucose could significantly stimulate insulin secretion in cells of all treatments (Fig. 1D). The stimulatory index (Fig. 1E), which reflects the elevation of stimulated secretion over the basal secretion, was also similar in different cell treatments. Furthermore, the expression level of the insulin1 gene did not change in cells, where FABP3 was knocked down (Fig. 1F).

The effect of FABP3 on FA-stimulated insulin secretion was examined (Fig. 1G) by exposure of *Fabp3*-down knocked and overexpressing Ins1E cells to ascending palmitate concentrations in the presence of a constant medium glucose concentration (11.1 mmol/L). FABP3 gene overexpression significantly enhanced the stimulatory effect of palmitic acid, whereas its silencing significantly reduces this stimulatory effect. In most palmitate concentrations, this stimulatory effect was blunted since no significant difference was observed in palmitate-stimulated secretion when compared with the value in the absence of palmitate.

Taken together, these results indicate that FABP3 plays a role in GSIS only in the presence of fatty acids. Also, the insulin stimulatory effect of FAs can be blunted by the inhibition of FABP3.

### FABP3 silencing inhibits FA uptake into insulin-secreting cells

**Fig. 2:**
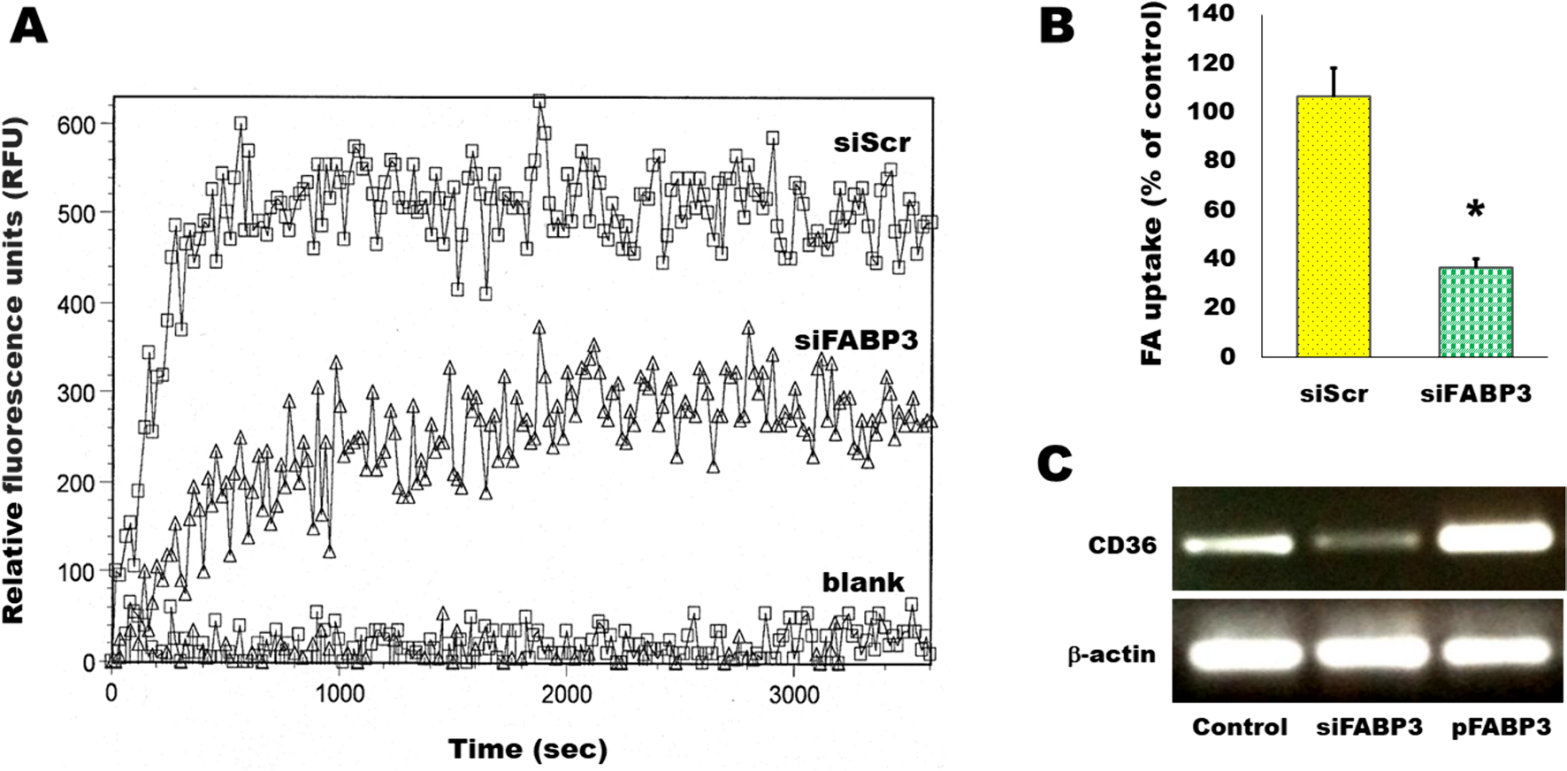
Silencing FABP3 reduces fatty acid uptake in insulin-secreting cells. **A** Time course of reduction of FA uptake by FABP3-gene silenced Ins1E cells, in comparison to the siScr control. **B** End-point result of fatty acid uptake by FABP3-down knocked Ins1E cells (*p < 0.05, unpaired *t*-test). **C** The silencing of FABP3 in Ins1E cells was accompanied by a decrease in the expression of the plasma membrane fatty acid receptor CD36 as detected by PCR

Ins1E cells were transfected by siRNA to knock down FABP3. These cells were incubated with fluorescent fatty acid and the kinetics of the intracellular up-taken fluorescence was read by fluorescence plate reader (Fig. 2A). Silencing of the FABP3 (siFABP3) gene reduced fatty acid uptake, compared with the corresponding siScr control uptake. The blank result shown in the figure shows the background fluorescence of cell-free wells. The endpoint FA uptake of the same cells after 1h of incubation with fluorescent lauric acid was significantly reduced by silencing of the FABP3 gene (Fig. 1B).

FAs are transported from the extracellular to the intracellular compartments by another protein: the cell membrane-bound fatty acid translocase CD36 [30]. The observation of decreased FA uptake by silencing FABP3 led us to explore the expression pattern of CD36 in these cells. Silencing FABP3 downregulated, while FABP3 overexpression upregulated CD36 expression, respectively (Fig. 2C). Therefore, it is not anticipated that CD36 intracellularly transfers extra fatty acids that are unable to be processed following FABP3 silencing. Thus, both FA transport across cell membrane and cellular uptake are decreased by *Fabp3* silencing in insulin-producing cells.

### Lipid accumulation in insulin-producing cells is inhibited by knocking down FABP3

**Fig. 3:**
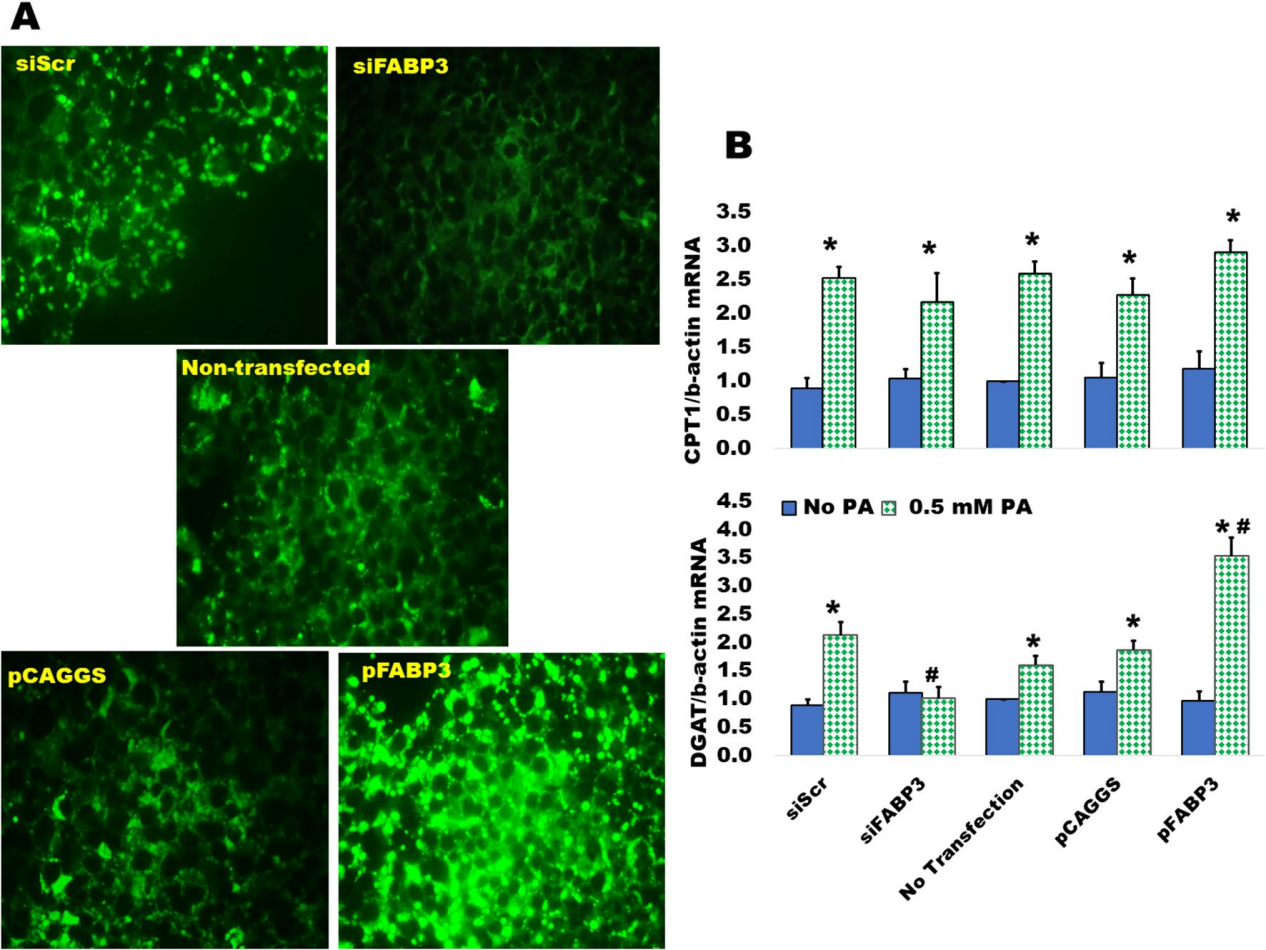
FABP3 regulated lipid accumulation in Ins1E cells. **A** FABP3 gene silencing reduced fat deposition in insulin-producing cells. Lipid droplets were detected in FABP3-silenced and overexpressing Ins1E cells incubated for 12 h with fluorescent lauric acid. **B** FABP3 silencing downregulated Dgat1 gene expression*.* CPT1a and Dgat1 gene expression were assessed by qRT-PCR of cells transfected for FABP3 silencing or overexpression and incubated for 24 h with 0.5 mmol/L palmitic acid. Statistical analysis: data are representative or means ± SEM of N = 3 experiments from different passages. The “*” = significantly different value from the “No palmitic acid” corresponding group value. The “#” denotes significantly different value from the corresponding controls (siScr and empty plasmid pCAGGS groups) (posthoc *t*-test after ANOVA)

The silencing of FABP3 gene inhibited lipid accumulation in Ins1E insulin-secreting cells, as compared to the siScr control, while FABP3 gene overexpression increased the accumulation of lipid droplets (Fig. 3A).

Fatty acid oxidation and triglyceride formation (lipid accumulation) are regulated by carnitine palmitoyltransferase 1a (CPT1a) and diacylglycerol acyltransferase 1 (Dgat1), respectively [8]. Both are upregulated by the influx of fatty acids. The expression of both genes was determined in FABP3-silenced and overexpressing Ins1E cells incubated with palmitic acid. Only Dgat1 expression was blunted in palmitate-stimulated FABP*3*-silenced cells (Fig. 3B). This effect was reversed in FABP3-overexpressing cells, indicating that FABP3 level controls Dagt1 expression and TG accumulation. FABP3 silencing did not affect the palmitate-induced CPT1a expression level, when compared to the siScr control value. This effect was similar in FABP3-overexpressing cells.

### FABP3 silencing inhibits FA-induced inflammatory response and apoptosis in insulin-producing cells

**Fig. 4:**
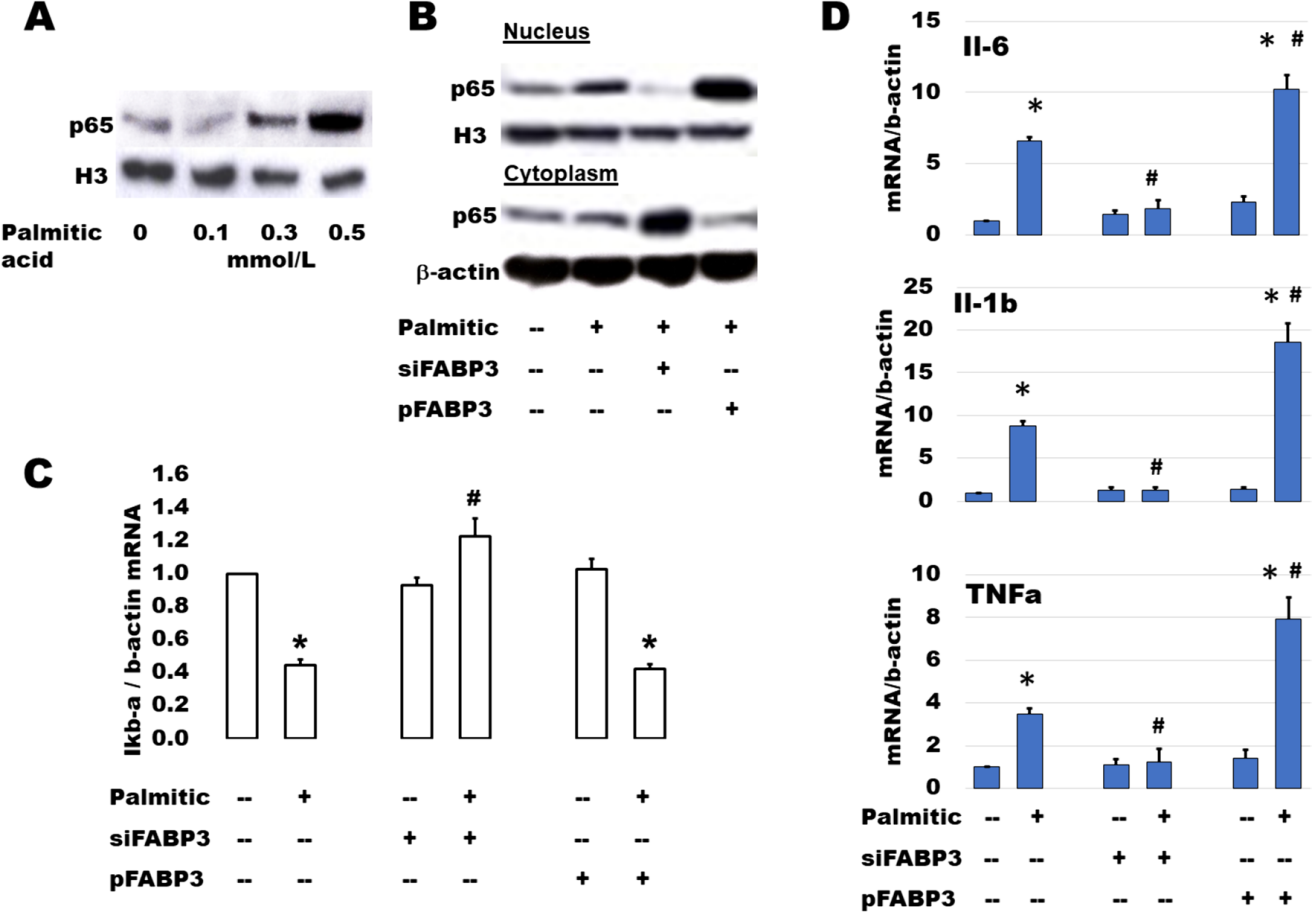
Inhibition of FABP3 reduced the lipid-induced inflammatory effect in insulin-secreting cells by inactivation of NF-κB. **A** Palmitic acid induced the NF-κB p65 (RelA) nuclear translocation in a FA-concentration-dependent manner. Western blot analysis of p65 in nuclear extract of Ins1E cells incubated with different concentrations of palmitic acid for 24 h. **B** FABP3 silencing inhibits, and overexpression provokes NF-κB activation. Cytoplasmic and nuclear proteins were extracted from transfected Ins1E cells and analyzed for p65 nuclear translocation by immunoblotting. **C** Silencing of FABP3 (siFABP3) upregulates and overexpression (pFABP3) downregulates IκBα expression in Ins1E cells as detected by qRT-PCR. **D** Downregulation of proinflammatory cytokine by silencing FABP3. Ins1E cells were transfected for FABP3 silencing or overexpression and incubated with 0.5 mmol/L palmitic acid for 24 h. Cytokine mRNA levels were detected by qRT-PCR. Statistical analysis: Data is presented as means ± SEM of N = 3 independent experiments for all subfigures. ANOVA *P* < 0.05. The “*” denotes a significantly different value from the corresponding control value. The “#” denotes significantly different value from the non-transfected palmitic acid-treated group value

Palmitic acid stimulates β-cell expression of main cytokines suspected of inducing β-cell dysfunction [31]. NF-κB plays a main role in palmitic acid-stimulated inflammation and β-cell failure [32]. The activation of NF-κB includes the translocation of p65 (RelA) protein from the cytosolic into the nuclear compartments via downregulation of IkBα. After incubation of Ins1E cells with different concentrations of palmitic acid for 24 h, p65 increased gradually with the increment of palmitic acid concentration (Fig. 4A). To study the effect of FABP3 silencing on the FA-induced inflammatory response, Ins1E cells were transfected for FABP3 silencing or overexpression and incubated with palmitic acid for 24 h. Silencing of FABP3 inhibited the p65 protein nuclear translocation. The reverse action was observed for FABP3 overexpression (Fig. 4B). Palmitic acid downregulated the expression of IkBα in control and FABP3-overexpressing cells, while FABP3 silencing hindered this downregulation (Fig. 4C). Taken together, NF-κB was not activated in FABP3-down knocked cells. Similar results were obtained for the expression of the proinflammatory cytokines Il-6, Il-1β, and TNFα (Fig. 1D). Incubation of Ins1E cells with palmitic acid upregulated the expression of these cytokines. This effect is increased in FABP3-overexpressing cells. In contrast, the expression of these inflammatory cytokines was not affected by palmitic acid in FABP3-silenced cells, and the values were significantly lower than that in the corresponding control (siScr-transfected cells).

**Fig. 5:**
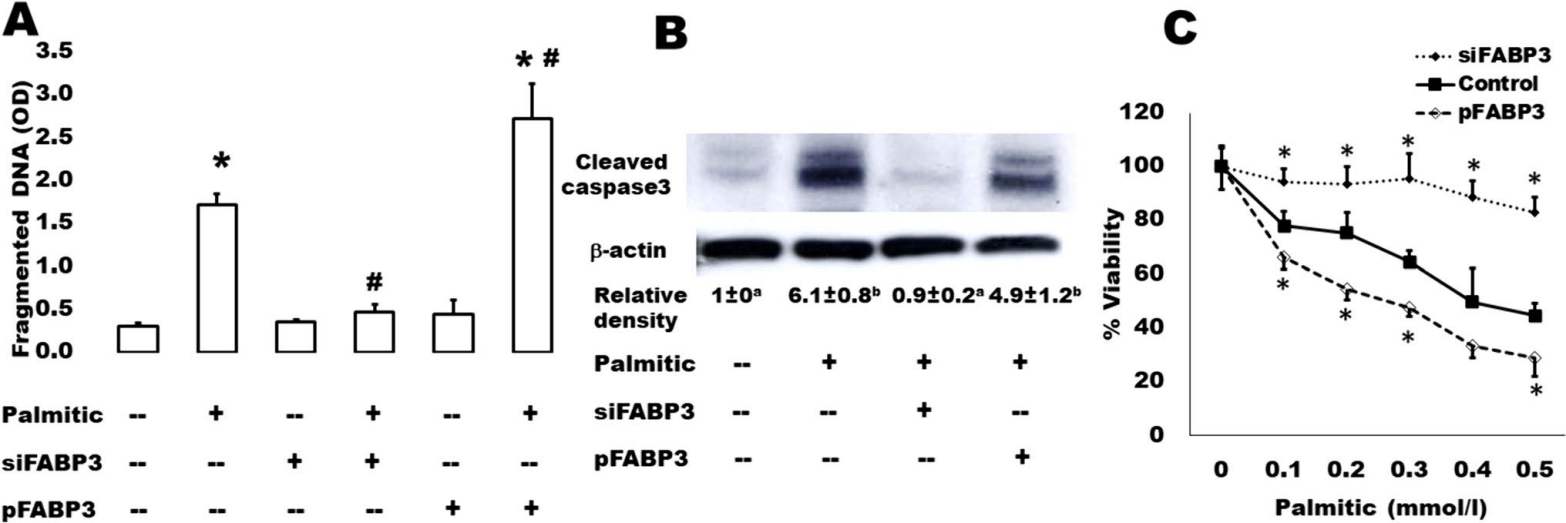
Protection of insulin-secreting cells against lipid-induced apoptosis and preservation of cell viability by FABP3 inhibition. **A** DNA fragmentation was measured using the cell death detection ELISA kit. INS-1E cells were transfected for overexpression (pFABP3) or silencing of FABP3 (siFABP3) and treated on the second day with 0.5 mmol/L of palmitic acid for 24 h. The medium contained 5.6 mmol/L glucose. Data are means ± SEM of 4 experiments from different passages. ANOVA *P* < 0.05; * denotes significantly (*p* < 0.05) higher OD value than the corresponding control value; # denotes significantly different value from OD value from non-transfected cells treated with palmitic acid (post hoc *t*-test). **B** Western blot analysis of cleaved caspase-3 protein from Ins1E cells of different treatments. Statistical analysis of relative densities: groups with different letters are significantly different from each other (N = 3). **C** Cell viability as assessed by MTT after culture with different palmitate concentrations for 24 h (N = 3). The “*” denotes a significantly different value from other corresponding values

We have further analyzed some apoptosis parameters in the same samples (Fig. 5). It is well-known that islets cultured in the presence of high levels of FAs exhibit the characteristic events of apoptosis including DNA fragmentation, increased caspase activity, ceramide formation, and expression of apoptotic genes [33]. The present findings demonstrate that this palmitate-induced effect on DNA fragmentation (Fig. 5A) and caspase 3 activation (Fig. 5B) significantly increased in FABP3-overexpressing Ins1E cells. In contrast, the FABP3-knocked down cells did not respond with apoptosis to exposure to palmitic acid: no changes were noticed regarding DNA fragmentation and cleaved caspase 3 in these cells. Cell death increased in directly proportional relationship with palmitic acid concentration (Fig. 5C). This could be inhibited by FABP3 silencing.

### Knocking down FABP3 maintained long-term insulin-secreting cell function

**Fig. 6:**
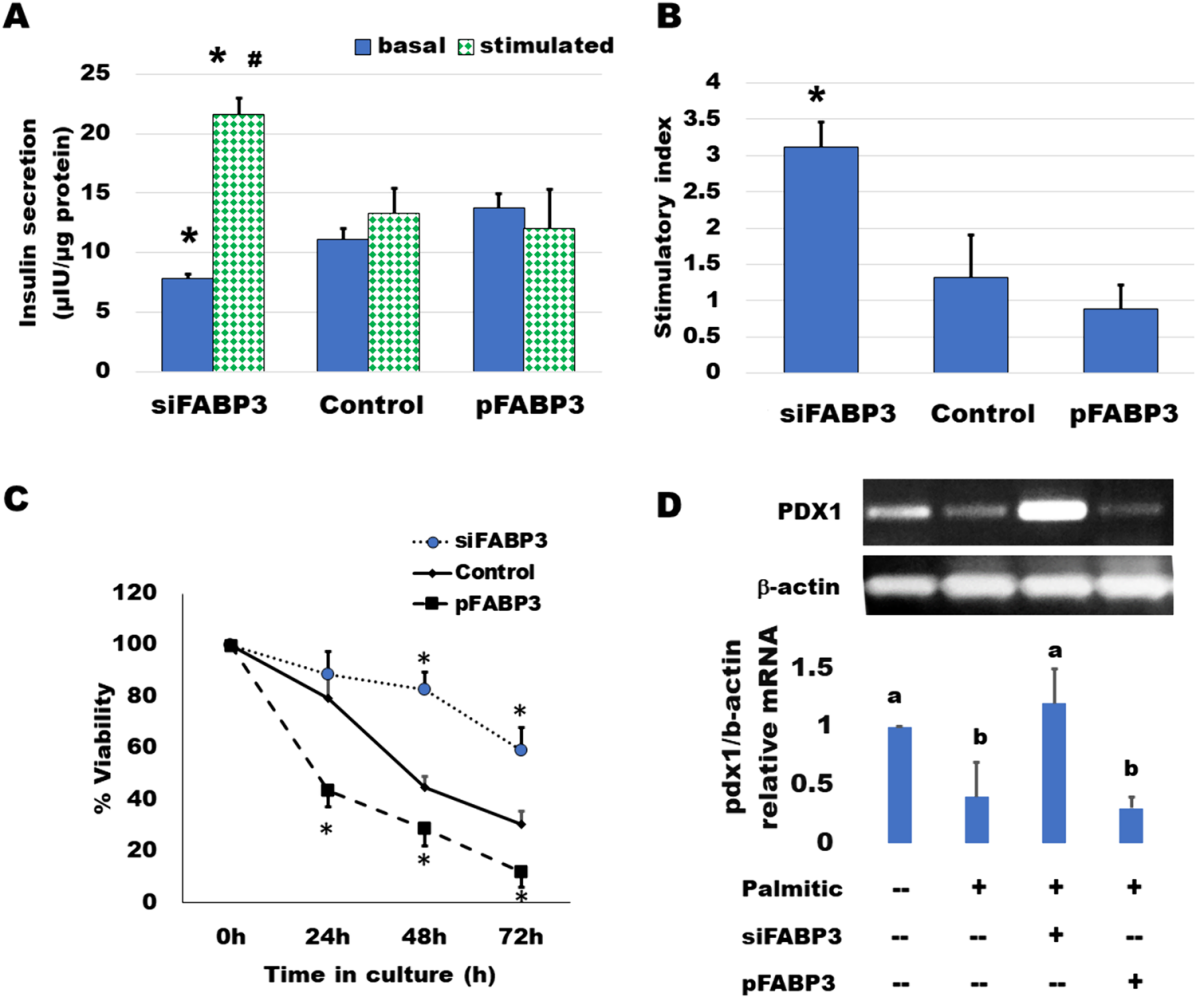
Improvement of long-term insulin-secreting cell function and viability by FABP3 silencing. **A** Basal and glucose-stimulated insulin secretion of FABP3-silenced or overexpressing Ins1E cells after 72 h culture in a medium containing 0.5 mmol/L palmitic acid. Data are presented as means ± SEM of N = 3. ANOVA. The “*” denotes significantly (*p* < 0.05) different value from the corresponding control, and the “#” denotes significantly different from the corresponding non-transfected cell value. **B** Stimulatory index is calculated as ratio between stimulated and basal insulin secretion. The “*” denotes significantly (*p* < 0.05) different value from the corresponding control, **C** Viability (N = 3) of FABP3-silenced or overexpressing Ins1E cells during the 72 h-culture in a medium containing 0.5 mmol/L palmitic acid. The “*” denotes a significantly different value from other corresponding values. **D** Gene expression of the determinant insulin transcriptional activator Pdx1 in FABP3-silenced or overexpressing Ins1E cells after 72 h-culture in a medium containing 0.5 mmol/L palmitic acid. For the RT-PCR, group values with different letters are significantly different from each other (N = 3)

Cells were transfected for silencing or overexpression of FABP3 and incubated to attach for 4 h. Then, the medium was replaced by that containing palmitate and incubated for 3 days. GSIS was found to be diminished by culture with 0.5 mmol/L palmitic acid (Fig. 6). Most cells looked dead and unattached. Glucose could not stimulate insulin secretion (Fig. 6A). The situation was similar in cultures of FABP3 overexpressing cells. However, in FABP3-silenced cells, the basal insulin secretion was significantly lower than that of both non-transfected and FABP3-overexpressing cells. The glucose-stimulated insulin secretion was significantly higher than its basal value and the value of non-transfected and overexpressing cells. The stimulatory index (Fig. 6B) was significantly higher in these FABP3-silenced cells than that of other treatments. The cell viability over the 3-day culture with palmitic acid (Fig. 6C) was maintained by knocking down FABP3 in comparison to non-transfected and FABP3-overexpressing cells. The functional β-cell-specific transcription factor PDX1 gene expression confirmed the results of insulin-secreting function in the same cultures (Fig. 6D). Knocking down FABP3 was found to significantly preserve PDX1 gene expression, while its expression decreased in other cultures treated with palmitic acid, as determined by qRT-PCR.

## Discussion

Lipotoxicity is a recognized mechanism of the loss of β-cell function in the pathophysiology of type 2 diabetes [34]. When entering β-cell, FAs mostly bind to fatty acid binding protein 3 to be metabolized. The main hypothesis in this work was: knocking down FABP3 in beta cells will reduce FA uptake and consequently the islet lipotoxicity and prevent islet cell apoptosis. Furthermore, inhibition of the fatty acid uptake by ß-cells might blunt the FA-induced inflammatory outcome [35]. This might contribute to improvement of beta cell survival and function, even in high fat conditions, and by that prevent or at least delay the incidence of diabetes.

The present study demonstrated that neither FABP3 inhibition nor overproduction affected the GSIS in the absence of FAs in culture medium. In contrast, in presence of stimulatory glucose concentration, there was a promotion in FA-induced insulin secretion from cells overexpressing FABP3, and a reduction of secretion from cells with down-knocked FABP3. We used a seemly high glucose concentration in this experiment because it is known that in beta cells lipotoxic effects are induced only in the presence of elevated glucose concentrations, since glucose is the determinant player in intracellular fatty acid destination [36, 37]. This finding minimizes the role of FABP3 in the absence of FAs and supports its role in the process of FA induction of insulin secretion. It also confirms the previous reports that, in contrast to glucose, exposure to FAs does not induce key changes in glucose-related gene expression in Ins1E cells and human islets [38]. This finding also indicates that inhibition of FABP3 may not be hazardous to beta cell glucose-stimulatory function.

The silencing of FABP3 gene in beta cells decreased both fatty acid uptake and the consequent fatty acid accumulation in insulin-secreting beta cells as proven by immunofluorescence. Also, it did not affect the expression of fatty acid transport and oxidation gene *Cpt1*, while it downregulated *Dgat1* and lipid accumulation. Defective fatty acid uptake was reported in several models of FABPs deficiency [7, 39–42]. A positive correlation exists between FABP levels and FA uptake [7]. In agreement with the decreased TG accumulation (lipid droplets) in *Fabp3*-knocked down cells, it was reported that in FABP3 KO mice, deficiency of FABP3 prevented the increase in cellular TG levels in skeletal muscle and heart that occur after long-term HFD [43].

Increasing the lipid availability and accumulation in beta cells was reported to induce many changes. It influences membrane and organelle structures, lowers membrane tension, and alters physicochemical properties of β-cells [44]; it significantly increases cellular ceramide levels that have been linked to pancreatic beta cell apoptosis and dysfunction [45]; and it induces apoptosis by stimulating the exhaustion of endoplasmic reticulum calcium stores, leading to the activation of inflammation and apoptotic pathway [46]. In the present study, FABP3 gene silencing caused a reduction of the FA availability and lipid accumulation inside the beta cells. As a result, our data revealed a reduction in inflammation and apoptosis in beta cells in response to fatty acids. The reduction of inflammation included lowering the expression of inflammatory cytokines, the inactivation of NF-κB, and the prevention of the deactivation of IkBa. The reduction of apoptosis included the inactivation of caspase 3 and prevention of DNA fragmentation. The mechanisms of beta cell inflammation and apoptosis include the activation of inflammatory signals like NF-κB, IL1b, IL-6, and TNFα, which impair insulin secretion. IL-1b activates the NF-κB signaling leading to dysregulation of FA metabolism and DNA fragmentation, resulting in beta cell dysfunction [47, 48]. The present results agree with these mechanisms and show that silencing of Fabp3 can minimize the lipid accumulation in beta cells and prevent the causative factors of inflammation and apoptosis. In other cell systems, and in agreement with our results, FABP3 deficiency also downregulated the expression of cleaved caspase 3 and Bax and upregulated the level of Bcl-2 in myocytes and exerted protective effects against ischemic heart injuries by decreasing cardiac myocyte apoptosis [13]. The present results agree with this protective role of FABP3 deficiency against beta cell apoptosis and dysfunction.

This protective role of FABP3 silencing was confirmed by a 72-culture with palmitate, and the data revealed normal Ins1E beta cells exhibiting normal viability, gene expression pattern, and insulin secretory function, despite the exposure to palmitic acid. The present data reveals that inhibition of FABP3 may protect pancreatic beta cells from lipotoxicity. In this context, we have reviewed some antidiabetic plants and found some natural products that can compete with FAs to bind FABP3 [20]. These competitive inhibitors might be safe to apply, but this remains to be elucidated. Other chemically designed commercial inhibitors are also available [49]

## Conclusion

Fatty acid binding protein 3 is produced in pancreatic beta cells to bind FAs and allow for FA metabolism. Long-term exposure of these cells to FA leads to inflammatory response, apoptosis, and dysfunction. In the present study, we have manipulated the expression of FABP3 by silencing or overexpression in Ins1E beta cells. Knocking down *Fabp3* was found to reduce FA uptake and lipid accumulation in these beta cells. This reduction protected cells from apoptosis and maintained their long-term function. FABP3 inhibitors may be useful to prevent the deleterious effects of FA binding and lipid accumulation in pancreatic islets resulting in ß-cell loss and contributing lastly to the pathogenesis of type 2 diabetes.

## Supplementary Information

Below is the link to the electronic supplementary material.

Supplementary file1 (DOCX 220 KB)

## Abbreviations

FA: Fatty acid
FABP: Fatty acid binding protein
GSIS: Glucose-stimulated insulin secretion
HFD: High-fat diet
Il: Interleukin
NF-κB: Nuclear factor kappa B
PUFA: Polyunsaturated fatty acid
TG: Triglycerides
TNFa: Tumor necrosis factor alpha
