# Supplementary material for: Silencing the FABP3 gene in insulin-secreting cells reduces fatty acid uptake and protects against lipotoxicity
Source: Acta Diabetol. 2024 Jul 4;61(12):1577–88. doi: 10.1007/s00592-024-02325-x (PMC11628584; doi:10.1007/s00592-024-02325-x)
Supplement: Supplementary file 1 — Supplementary file1 (DOCX 220 KB) [file 592_2024_2325_MOESM1_ESM.docx]

**Silencing the FABP3 gene in insulin-secreting cells reduces fatty acid uptake and protects against lipotoxicity**

Ayman Hyder^1*^, Basma Sheta^1^, Manar Eissa^1#^, Jürgen Schrezenmeir^2^

^1^Faculty of Science, Damietta University, New Damietta 34517, Egypt.

^2^Faculty of Medicine, Johannes Gutenberg University, Mainz, Germany

Supplementary data:


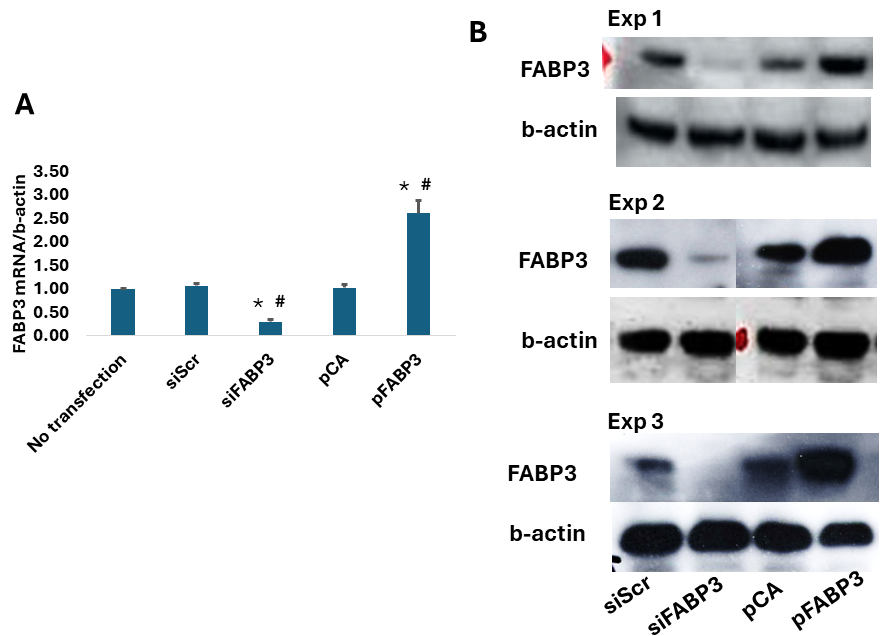


**Figure S1**. Efficiency of transfection. Ins1E cells were transfected as described in the Material & Methods section with either FABP3 silencer (siFABP3), silencing-scrambled control (siScr), FABP3 plasmid (pFABP3) for overexpression, or the empty plasmid (pCA) as a corresponding control. **A)** Quantitative RT-PCR of FABP3 mRNA expression after silencing/overexpression. Data is presented as means±SE of 3 independent transfection experiments. ANOVA p <0.0001. The “*” denotes a significantly different value from the corresponding control, and the “#” denotes a significantly different value from the value of the non-transfected group. **B)** Western blot analysis of FABP3 protein expression after silencing/overexpression. Data is the outcome of 3 independent experiments using Ins1E cells of different passages.

**Table S1**. Primer sequences of studied genes for RT-PCR

| Gene | Accession No. | Sense primer | Antisense primer | Product size (bp) |
| --- | --- | --- | --- | --- |
| actb | **NM_031144.3** | **5’-** **CACCCGCGAGTACAACCTTC-3’** | **5’-** **ACGTAGGAGTCCTTCTGACCC-3’** | **225** |
| Cd36 | **NM_031561.2** | **5’-CACAGATGCAGCCTCCTTTC-3’** | **5’-CAGTGGTTGTCTGGGTTCTG-3’** | **189** |
| Cpt1a | **NM_031559.2** | **5’-AGAGAGGAGGATCCTGAGGC-3’** | **5’-GGTGGCCATGACATACTCCC-3’** | **192** |
| Dgat1 | **NM_053437.2** | **5’-TCTACAGGGACTGGTGGAATG-3’** | **5’-CTCAGGGGAATGCTCACTAGG-3’** | **193** |
| Fabp3 | **NM_024162.2** | **5’-CGGTACCTGGAAGCTAGTGG-3’** | **5’-TCATCTGCTGTGACCTCGTC-3’** | **216** |
| Ikba | **NM_001105720.2** | **5’-TCACGGAAGATGAGTTGCCC-3’** | **5’-ACCACAATAGAATGCTCGGGG-3’** | **213** |
| Il1b | **NM_031512.2** | **5’-GCAGCTTTCGACAGTGAGGA-3’** | **5’-CTCCACGGGCAAGACATAGG-3’** | **215** |
| Il6 | **NM_012589.2** | **5’-TCTCCGCAAGAGACTTCCAG-3’** | **5’-TTGCCATTGCACAACTCTTTTC-3’** | **208** |
| Insulin1 | **NM_019129.3** | **5’-AGGCTCTGTACCTGGTGTGT-3’** | **5’-AGTTGGTAGAGGGAGCAGATG-3’** | **215** |
| Pdx1 | **NM_022852.4** | **5’-AAAAGCCAGTGGGCAGGAGG-3’** | **5’-TTCATGCGACGGTTTTGGAAC-3’** | **209** |
| Tnfa | **NM_012675.3** | **5’-TTCTCATTCCTGCTCGTGGC-3’** | **5’-TCCGCTTGGTGGTTTGCTAC-3’** | **200** |

Selected primers span exon-exon junction. Annealing temperature in PCR programming is 60^o^C for all gene
